# Long-term survival of a patient with recurrent gallbladder carcinoma, treated with chemotherapy, immunotherapy, and surgery: a case report

**DOI:** 10.1186/s40792-018-0512-6

**Published:** 2018-09-15

**Authors:** Makoto Kawamoto, Yoshiyuki Wada, Norihiro Koya, Yuko Takami, Hideki Saitsu, Naoki Ishizaki, Mineo Tabata, Hideya Onishi, Masafumi Nakamura, Takashi Morisaki

**Affiliations:** 1 Department of Cancer Therapy and Research, Graduate School of Medical Sciences, Kyushu University, Fukuoka, Japan; 2 Department of Hepato-Biliary-Pancreatic Surgery, National Hospital Organization Kyushu Medical Center, Fukuoka, Japan; 3 Fukuoka General Cancer Clinic, 3-1-1 Sumiyoshi, Hakata-ku, Fukuoka, 812-0018 Japan; 4 Department of Surgery, Kagoshima Medical Association Hospital, Kagoshima, Japan; 5 Departments of Surgery and Oncology, Graduate School of Medical Sciences, Kyushu University, Fukuoka, Japan

**Keywords:** Immunotherapy, Cytokine-activated killer cell, NKG2D, Gallbladder cancer, MUC-1

## Abstract

**Background:**

Gallbladder cancer (GBC) is one of the refractory diseases. Multidisciplinary approach including immunotherapy for such cancers has received much attention in recent years.

**Case presentation:**

A 59-year-old man underwent an extended cholecystectomy for GBC (pathological stage II, T2 N0 M0, [per UICC 7th edition]) that was incidentally found during cholelithiasis surgery, and was then treated with adjuvant gemcitabine (GEM). Three months later, when a recurrence-suspected lesion was detected in segment 5 (S5) of his liver, we started adoptive immunotherapies with cytokine-activated killer (CAK) cell infusions, combined with chemotherapy. After a year of adjuvant immunochemotherapy, the S5 lesion disappeared on imaging, but lesions suspected metastatic recurrence again appeared in S7 and S8 at 4 years and 6 months post-surgery, for which GEM and cisplatin (CDDP) were administered as second-line chemotherapy. Immunochemotherapy produced stable disease (per RECIST) for 9 months, when tumor growth was detected; open microwave coagulo-necrotic therapy (MCN) was performed for these lesions. Three years after MCN, a solitary liver metastasis was detected in S4. MCN was conducted again, and peritoneal dissemination was found intraoperatively. A month after the second MCN, the patient’s carcinoembryonic antigen (CEA) level had increased. Therefore, GEM and tegafur-gimeracil-oteracil potassium (TS-1) were administered as third-line chemotherapy. We also switched the adoptive immunotherapy for tumor-associated antigen-pulsed dendritic cell-activated killer (DAK) cell immunotherapy. After nine courses of GEM and TS-1 administration, CEA had decreased to a normal level. At the time of reporting, 9 years and 6 months have passed since the initial surgery, and 18 months have passed since the peritoneal metastasis was detected. GEM and CDDP are currently administered as fourth-line chemotherapy because of re-increased CEA. Although an undeniable metastasis was found in his para-aortic lymph node, this patient visits our clinic regularly for immunotherapy.

**Conclusion:**

We here report a rare case of long-term survival of recurrent GBC well controlled by multidisciplinary therapy. Immunotherapy may be a promising modality among multidisciplinary methods for advanced cancer.

## Background

Gallbladder cancer (GBC) is usually a fatal disease. Although complete resection is the only potentially curative treatment, most GBC cases will have developed into locally advanced disease or have metastasized by the time of diagnosis. In inoperable cases, many patients must rely on chemotherapy and radiation therapy, which are not sufficiently effective.

Multidisciplinary treatments for advanced cancers that include immunotherapy have received much attention in recent years [1]. Many patients with advanced cancer cannot receive long-term chemotherapy because the adverse effects of the treatment are not tolerable to such patients. In this situation, immunotherapy could be a reliable candidate to improve the prognosis of these patients without lowering their quality of life.

We report here a rare case of a patient who has currently survived almost 10 years with recurrent GBC with peritoneal dissemination and liver metastases, which has been well controlled by a multidisciplinary approach including chemotherapy, immunotherapy, and surgery.

## Case presentation

**Fig. 1:**
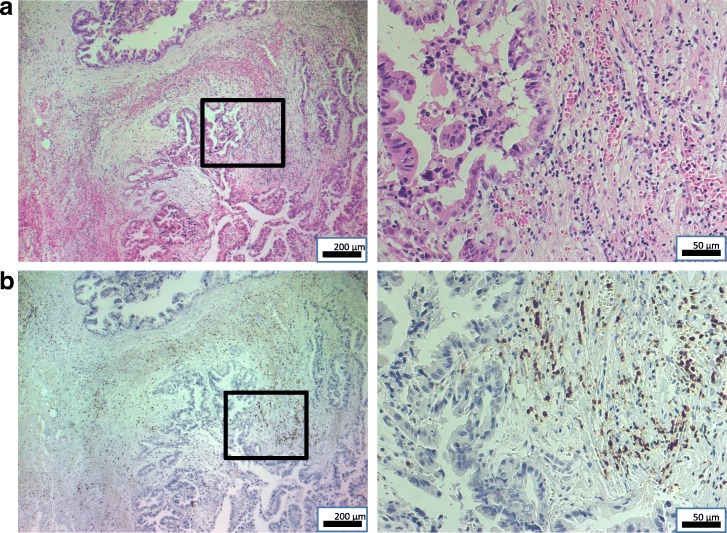
Representative hematoxylin–eosin-stained images and CD3^+^ immunohistochemistry results in primary gallbladder cancer specimen. **a** Specimen with tumor-infiltrating lymphocytes. Right: × 200; left (insert): × 50. **b** Lymphocytes infiltrate tumor stroma. Brown chromogen: CD3^+^ T cells. Right: × 200; left (insert): × 50

A 59-year-old Japanese man was referred to hospital with right upper quadrant pain. He underwent laparoscopic cholecystectomy on the diagnosis of cholelithiasis. However, because intraoperative pathological diagnosis revealed GBC, we performed an extended cholecystectomy that included resections of the gallbladder bed and extrahepatic bile duct, and D2 lymphadenectomy, with choledochojejunostomy reconstruction. The pathological diagnosis was well-differentiated adenocarcinoma of the gallbladder, T2 N0 M0, stage II (Union for International Cancer Control, 7th edition) (Fig. 1).

**Fig. 2:**
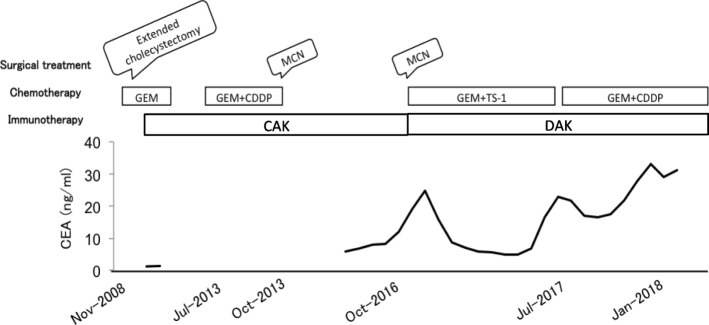
CEA levels throughout the entire treatment course

**Fig. 3:**
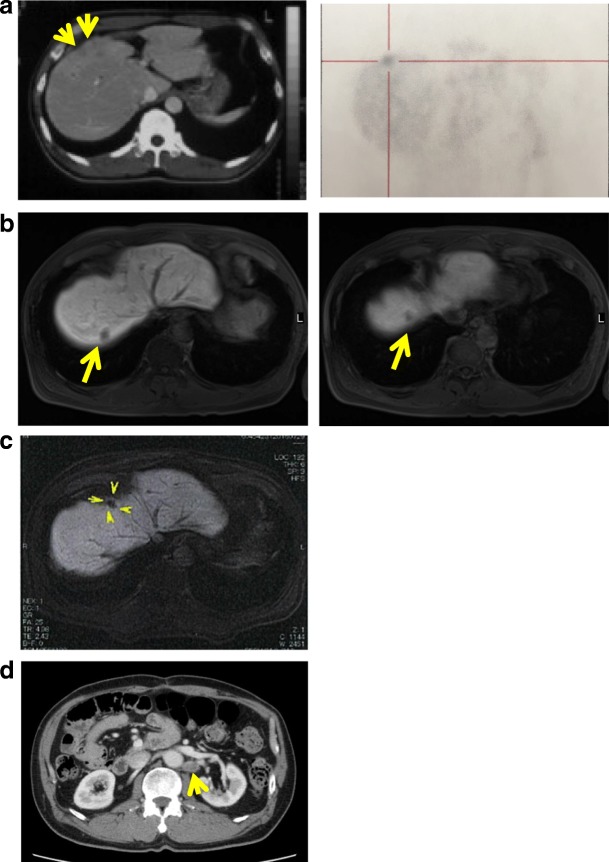
Image diagnosis. **a** Left: positron emission tomography-computed tomography (PET-CT) findings at 3 months after surgery shows poor contrast enhancement area in S5 (arrows). Right: abnormal ^18^F-fluorodeoxyglucose (FDG) uptake was detected in the same lesion. **b** Magnetic resonance imaging (MRI) with gadolinium ethoxybenzyl diethylenetriamine penta-acetic acid (Gd-EOB-DTPA) before first microwave coagulo-necrotic therapy (MCN) shows hypointense lesions (arrows) at S7 and S8 in the hepatobiliary phase. **c** MRI with Gd-EOB-DTPA before second MCN shows hypointense lesions at S4 (arrows) in the hepatobiliary phase. **d** Current computed tomography image shows undeniable metastasis (arrow) with contrast effect in para-aortic lymph node, but no ascites

The patient’s clinical course and associated tumor makers are illustrated in Fig. 2. He was treated with adjuvant gemcitabine (GEM). GEM (1600 mg/body) was administered weekly, three times every 4 weeks. Three months after surgery, abnormal ^18^F-fluorodeoxyglucose (FDG) uptake was detected in segment 5 (S5) of the patient’s liver (Fig. 3a), which suggested metastatic recurrence. We commenced adoptive immunotherapies with cytokine-activated killer (CAK) cell infusions at our clinic, combined with chemotherapy. After a year of adjuvant chemotherapy and immunotherapy, the S5 lesion had disappeared on FDG-PET.

**Fig. 4:**
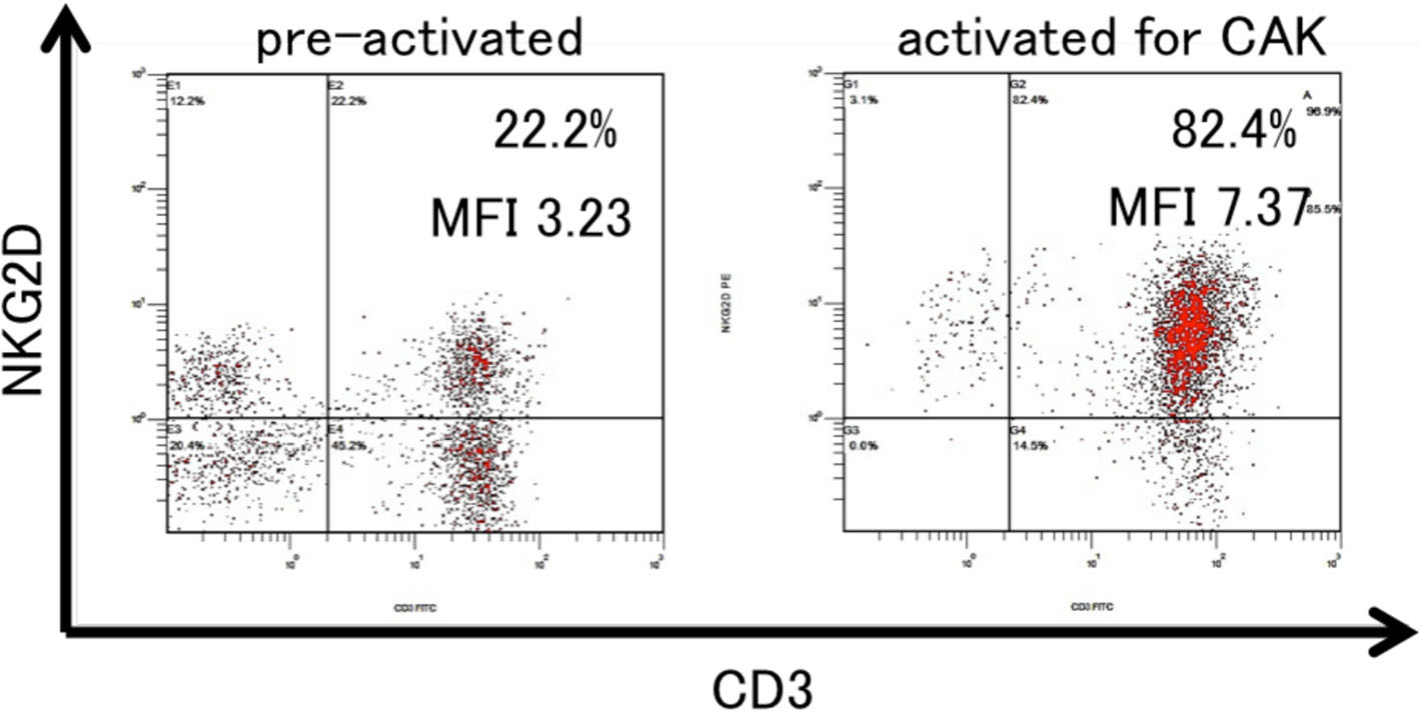
Representative histogram of natural-killer group 2, member D (NKG2D) expression on lymphocytes, before and after stimulation. NKG2D expression levels on lymphocytes before and after activation were analyzed by fluorescence-activated cell sorting. Activation notably increased NKG2D expression on CAK cells

CAK cells consist of activated T cells that express high levels of the activating receptor, natural-killer group 2, member D (NKG2D), and activated natural killer (NK) cells (Fig. 4). The procedure for CAK cell generation has been described previously [2, 3]. Briefly, peripheral blood mononuclear cells (PBMCs) were collected with a blood cell separator (Haemonetics CCS, Haemonetics Corporation, Braintree, MA, USA) and cryopreserved until use. PBMCs were stimulated with both human recombinant interleukin (IL)-2 (rIL-2, 200 U/ml; Primmune Inc. Kobe, Japan) and 5 μg/ml antibody to CD3 (MACS GMP CD3 pure; Miltenyi Biotec Inc. Auburn, CA, USA). After 7 days in culture, PBMCs were transferred to a culture bag system (KBM550, Kohjin Bio, Osaka, Japan) and expanded for 7 days to obtain sufficient numbers of CAK cells.

At 4 years and 6 months post-surgery, contrast-enhanced magnetic resonance imaging (MRI) showed low-signal lesions in S7 and S8 in the hepatobiliary phase. ^18^F-fluorodeoxyglucose positron emission tomography (FDG-PET) revealed abnormal FDG uptake in the same lesions detected by MRI (Fig. 3b), which suggested metastatic recurrence. Therefore, GEM and cisplatin (CDDP) were administered as second-line chemotherapy. GEM (1600 mg/body) and CDDP (40 mg/body) were administered intravenously on days 1 and 8, followed by a week rest period. The patient had a stable disease response to chemotherapy and immunotherapy for 9 months (as defined by the Response Evaluation Criteria in Solid Tumors). However, because the lesions increased in size again, we performed open microwave coagulo-necrotic therapy (MCN) for the S7 and S8 lesions. No peritoneal dissemination or liver metastasis was detected during this procedure.

**Fig. 5:**
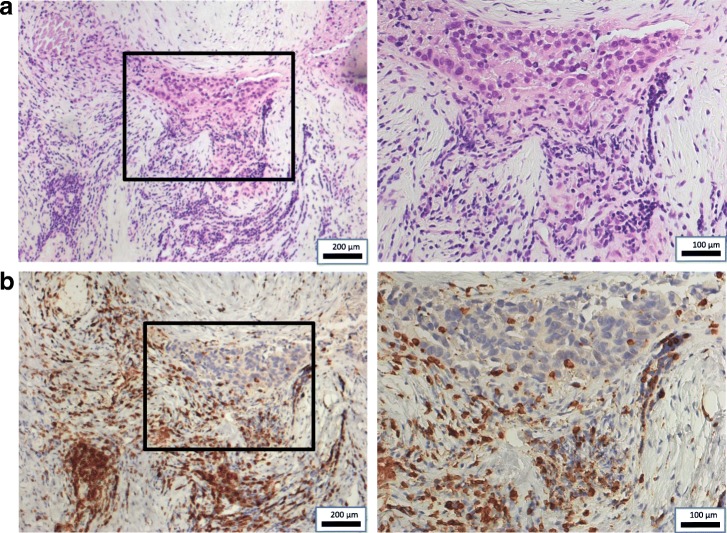
Representative hematoxylin–eosin-stained images and CD3^+^ immunohistochemistry results in disseminated nodule specimen. **a** Tumor cells and tumor-infiltrating lymphocytes. Right: × 200; left (insert): × 100. **b** Rich lymphocytic infiltration around tumor cells. Brown chromogen: CD3^+^ T cells. Right: × 200; left (insert): × 100

Three years after the initial MCN, a solitary liver metastasis was detected at the S4 liver surface by contrast-enhanced MRI (Fig. 3c), for which MCN was conducted again in consideration of the solitary lesion being in the liver surface. During this procedure, peritoneal seeding was found and intraoperatively diagnosed as adenocarcinoma, indicating peritoneal GBC dissemination. Histopathological examination of the disseminated nodule specimens showed many infiltrating mononuclear CD3^+^ cells around the tumor cells (Fig. 5). A month after the second MCN, the patient’s carcinoembryonic antigen (CEA) level had increased to 24.8 ng/ml, although imaging showed no recurrence. Therefore, GEM and tegafur-gimeracil-oteracil potassium (TS-1) were administered as third-line chemotherapy. GEM (1600 mg/body) was administered intravenously on day 1, and TS-1 (120 mg/day) was administered orally for 2 weeks, followed by a week rest period. After nine cycles of GEM and TS-1, the CEA had decreased to a normal level.

**Fig. 6:**
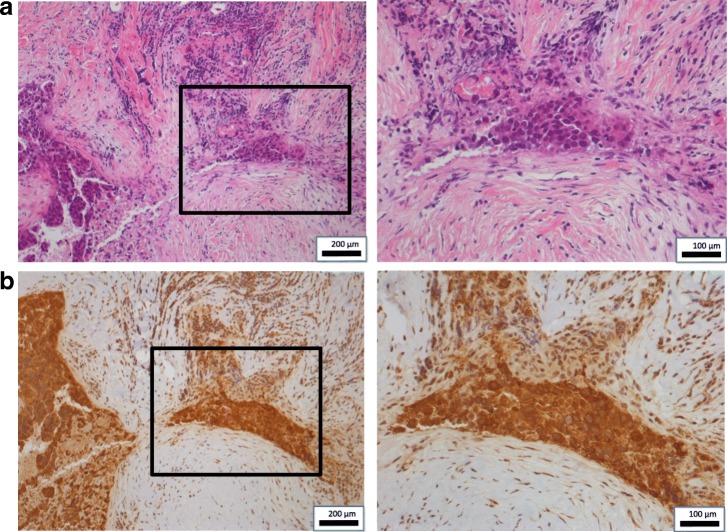
Representative hematoxylin–eosin-stained images and MUC-1 immunohistochemistry results in disseminated nodule specimen. **a** Disseminated tumor cells. Right: × 200; left (insert): × 100. **b** MUC-1 expression of disseminated tumor cells. Brown chromogen: MUC-1^+^ cells. Right: × 200; left (insert): × 100

For second-line adoptive immunotherapy, we used adoptive infusions of tumor-associated antigen-pulsed dendritic cell-activated killer (DAK) cell immunotherapy. Because immunohistochemistry showed MUC-1 positive cells in the disseminated tumors (Fig. 6), we chose long MUC-1 peptides for the tumor-associated antigens. For the DAK cell culture, we prepared immature dendritic cells from plastic-adherent PBMCs, using recombinant human granulocyte/monocyte colony-stimulating factor (GM-CSF; 100 ng/ml; Primmune Inc.) and recombinant human IL-4 (500 U/ml; Primmune Inc.) for 7 days. MUC-1 long peptides (Miltenyi Biotec, Bergisch Gladbach, Germany) were added to immature dendritic cells at a final concentration of 50 nmol/ml, then incubated for 12 h following by addition of human recombinant tumor necrosis factor-α (TNF-α, 500 U/ml; Primmune Inc.) and interferon-α (IFN-α; 500 U/ml; MSD Inc. Tokyo, Japan). Autologous lymphocytes were then added to the dendritic cells at a ratio of 20:1 (lymphocytes:dendritic cells) and cultured for 7 days. The cells were then activated and expanded with IL-2 and antibody to CD3 for 14 days, as with the CAK cell culture.

Over the past 9 years, we have performed adoptive cell therapy procedures 59 times, including 38 CAK cell infusions and 21 MUC-1-pulsed DAK cell infusions, at 1–3-month intervals, along with chemotherapy. All the cultures we used were inspected for contamination with endotoxin, β-glucan, and peptide-glycan using a Toxinometer ET-6000 (Wako Pure Chemical Industries, Ltd., Osaka Japan), according to Food and Drug Administration guidelines. Mycoplasma contamination was checked using a Mycoalert kit (Lonza Rockland Inc., ME, USA).

The cell processing and adoptive immunotherapy procedures were approved by the ethics committee of our institution with the patient’s written informed consent for the procedure, based on the Act on Securement of Safety of Regenerative Medicine in Japan.

At the time of reporting, 9 years and 6 months have passed since the initial cholecystectomy, and 18 months have passed since the peritoneal metastasis was detected. At this time, the patient is receiving GEM and CDDP as fourth-line chemotherapy because of increased CEA levels. Although an undeniable metastasis was found in his para-aortic lymph node (Fig. 3d), the patient visits our clinic regularly for an immunotherapy. His only immunotherapy-related adverse event was a low-grade fever that did not noticeably lower his quality of life.

### Discussion

The reported 5-year survival rates of patients with GBC are stage 0, 80%; stage I, 50%; stage II, 28% [4]; and stages III–IV, < 10% [5]. Reportedly, 47% of the GBC cases are discovered incidentally during laparoscopic cholecystectomy, as in this case [6]. Therefore, careful histopathological review should follow surgery for cholelithiasis. The 5-year survival for incidental GBC has been shown to increase to 80% when patients undergo curative surgery [7].

Complete resection is the only potentially curative treatment for GBC. However, even in cases of metastatic GBC, resection might lead to long-term survival if the metastatic lesions are resectable and well controlled with other therapies [8]; there was no standard surgical treatment that has been established for recurrent GBC. Then, we chose MCN as part of multidisciplinary therapy. The main chemotherapeutic agents for GBC are GEM, CDDP, and 5-FU, alone or in combination. The combination of GEM and CDDP for unresectable patients elicited response rates of 21 to 48%, with median survival times (MST) of 4.6 months to 11 months in several reports [9–12]. As these responses are not satisfactory, and the benefits of adjuvant chemotherapy for GBC are unclear, multidisciplinary therapy is required for patients with GBC.

Immunotherapy may be a promising modality among multidisciplinary treatments for far-advanced cancers. Shimizu et al. reported a survival benefit associated with postoperative adjuvant immunotherapies that included autologous tumor lysate-pulsed dendritic-cell vaccine and activated T cell transfer among patients with intrahepatic bile duct cancer [13]. Kimura et al. also reported that adjuvant immunotherapies improved postsurgical prognosis in a randomized controlled, phase III trial of patients with lung cancer [14]. Yang et al. reported that DC-activated, cytokine-induced killer cells enhanced the anti-tumor effects of chemotherapy in patients with advanced non-small cell lung cancer [15]. We also previously reported on the synergistic effects of gemcitabine and CAK cell immunotherapy [2]. The numbers of tumor-infiltrating lymphocytes have also been reported to be a prognostic factor [16].

Because there was no standard treatment that has been established other than GEM at that time, we used immunotherapy combined with GEM. There was no significant chemotherapy-related adverse event in the first GEM and CDDP therapy. Therefore, we chose GEM and CDDP therapy as fourth-line chemotherapy combined with second-line immunotherapy, expecting for their synergistic effects.

The CAK cells used in the current case were a heterogeneous population that included activated NK cells and T cells, which are important tumor-antigen-independent effectors of immune response to tumors [2]. Because NKG2D is an activating receptor found on activated NK and activated T cells, high NKG2D expression was used to characterize the quality of CAK cells used for infusion. Our earlier report and other studies showed that the strength of the anti-tumor immune response depended on surface levels of NKG2D [17–19]. CAK cells are thought to have affected tumor repression in the current patient. We considered CD3^+^ tumor-infiltrating lymphocytes detected in the resected disseminated nodules to reflect this.

The second-line adoptive immunotherapy that we used was DAK cell therapy. Many studies have reported the efficacy and safety of tumor-associated, antigen-pulsed, dendritic cell-activated lymphocyte therapy [20–22]. MUC-1 expression was confirmed by immunostaining a sample of the disseminated tumor, which encouraged us to use MUC-1 long-peptide vaccine as the tumor-associated antigen. MUC-1 has been shown to be a tumor-associated antigen and target molecule for dendritic cell-based immunotherapy in advanced biliary tract cancer [23, 24].

At present, the patient visits our clinic regularly for immunotherapy. He has suffered no apparent immunotherapy-related side effect except for low-grade fever. Since there are few reports about topical treatments for the recurrent GBC, we suppose this case would be valuable to assess the significance of the surgical treatments including MCN for the recurrent GBC. Although distinguishing between the contributions of chemotherapy and immunotherapy to his clinical response is not possible, the present case nevertheless suggests the auxiliary effects of immunotherapy for far-advanced cancers.

## Conclusions

We reported a rare case of long-term survival of recurrent GBC that was well controlled by multidisciplinary therapy, including chemotherapy, immunotherapy, and surgery. This approach thus deserves further investigation to confirm its value in patients with far-advanced cancers.

## Abbreviations

CAK: Cytokine-activated killer
CEA: Carcinoembryonic antigen
DAK: Dendritic cell-activated killer
FDG-PET: ^18^F-fluorodeoxyglucose positron emission tomography
GBC: Gallbladder cancer
IFN-α: Interferon-α
MCN: Microwave coagulo-necrotic therapy
MRI: Magnetic resonance imaging
MST: Median survival time
NK: Natural killer
NKG2D: Natural-killer group 2, member D
PBMCs: Peripheral blood mononuclear cells
RR: Response rate
TNF-α: Tumor necrosis factor-α
